# Genome-centric view of the microbiome in a new deep-sea glass sponge species *Bathydorus* sp.

**DOI:** 10.3389/fmicb.2023.1078171

**Published:** 2023-02-08

**Authors:** Tao-Shu Wei, Zhao-Ming Gao, Lin Gong, Qing-Mei Li, Ying-Li Zhou, Hua-Guan Chen, Li-Sheng He, Yong Wang

**Affiliations:** ^1^Institute of Deep-Sea Science and Engineering, Chinese Academy of Sciences, Sanya, Hainan, China; ^2^University of Chinese Academy of Sciences, Beijing, China; ^3^Institute of Oceanology, Chinese Academy of Sciences, Qingdao, Shandong, China; ^4^Institute for Ocean Engineering, Shenzhen International Graduate School, Tsinghua University, Shenzhen, China

**Keywords:** ammonia-oxidizing archaea, symbiont *Bdellovibrio*, the South China Sea, CRISPR, phage

## Abstract

Sponges are widely distributed in the global ocean and harbor diverse symbiotic microbes with mutualistic relationships. However, sponge symbionts in the deep sea remain poorly studied at the genome level. Here, we report a new glass sponge species of the genus *Bathydorus* and provide a genome-centric view of its microbiome. We obtained 14 high-quality prokaryotic metagenome-assembled genomes (MAGs) affiliated with the phyla Nitrososphaerota, Pseudomonadota, Nitrospirota, Bdellovibrionota, SAR324, Bacteroidota, and Patescibacteria. In total, 13 of these MAGs probably represent new species, suggesting the high novelty of the deep-sea glass sponge microbiome. An ammonia-oxidizing Nitrososphaerota MAG B01, which accounted for up to 70% of the metagenome reads, dominated the sponge microbiomes. The B01 genome had a highly complex CRISPR array, which likely represents an advantageous evolution toward a symbiotic lifestyle and forceful ability to defend against phages. A sulfur-oxidizing Gammaproteobacteria species was the second most dominant symbiont, and a nitrite-oxidizing Nitrospirota species could also be detected, but with lower relative abundance. *Bdellovibrio* species represented by two MAGs, B11 and B12, were first reported as potential predatory symbionts in deep-sea glass sponges and have undergone dramatic genome reduction. Comprehensive functional analysis indicated that most of the sponge symbionts encoded CRISPR–Cas systems and eukaryotic-like proteins for symbiotic interactions with the host. Metabolic reconstruction further illustrated their essential roles in carbon, nitrogen, and sulfur cycles. In addition, diverse putative phages were identified from the sponge metagenomes. Our study expands the knowledge of microbial diversity, evolutionary adaption, and metabolic complementarity in deep-sea glass sponges.

## Introduction

Marine sponges in the phylum Porifera are important members of marine benthic communities and emerged on Earth at ~600 mya (Yin et al., 2015). Sponges are extensively found throughout the global oceans, from shallow water to deep sea, from temperate to arctic regions, along shelves, on ridges, and on seamounts (Howell et al., 2016; Maldonado et al., 2017). Sponges usually harbor dense and diverse prokaryotic communities, which can account for up to 35% of the sponge biomass (Webster and Thomas, 2016; Pita et al., 2018). In terms of taxonomic diversity, up to 63 prokaryotic phyla have been recovered from marine sponges (Schmitt et al., 2012; Moitinho-Silva et al., 2017b). Considering the diversity and potential functional importance of the symbiotic microbiome, sponges are frequently referred to “holobionts”, a complex and interdependent consortium that comprises the sponge host and the entire microbiome (Pita et al., 2018). Because of their ancient origin and intense association with microbes, sponges and their microbiomes from shallow waters have been extensively studied (Thomas et al., 2016; Zhang et al., 2019a).

Deep-sea sponges are of importance in ecology functions such as microhabitat provision, substrate modification, benthic–pelagic coupling, and nutrient cycling (Bell, 2008; Maldonado et al., 2012; de Goeij et al., 2013; Kutti et al., 2013; Hawkes et al., 2019). The diversity and novelty of deep-sea sponges have drawn increasing attention, as well as the associated microbiomes (Steinert et al., 2020; Busch et al., 2022). Nutrient conversions by the ammonia-oxidizing archaea (AOA), the nitrite-oxidizing bacterium (NOB), and the sulfur-oxidizing bacterium (SOB) inhabiting the deep-sea glass sponge *Lophophysema eversa* were revealed to carry out relatively complete carbon, sulfur, and nitrogen cycles (Tian et al., 2016). The major microbial lineages in the deep-sea glass sponge *Vazella pourtalesii* underlined probably benefit from their small genome sizes and low GC contents likely due to adaptation to the unique seawater environment (Bayer et al., 2020). Especially, a comprehensive analysis of prokaryotic communities associated with 13 phylogenetically diverse deep-sea sponge species in the South Pacific Ocean revealed that archaeal 16S rRNA gene numbers were up to three orders of magnitude higher than those in shallow-water sponges, which highlighted the importance of the archaea for deep-sea sponges (Steinert et al., 2020). A large-scale analysis of microbial diversity further revealed the biodiversity, environmental drivers, and sustainability of the global deep-sea sponge microbiome, and underscored the uniqueness of each deep-sea sponge ground (Busch et al., 2022). However, knowledge about the diversity and novelty of deep-sea sponge microbial consortia remains limited. The metabolic potential and mutualistic strategies of deep-sea sponge symbionts are also poorly studied.

Following the deepening research of sponge symbionts, viruses in sponges and their functions on the sponge holobionts have also drawn increasing attention recently. Viruses are the most abundant organisms in marine environments, infecting nearly all organisms and having a direct impact on energy flux in marine food webs by regulating prokaryotic and eukaryotic populations (Suttle, 2005; Roux et al., 2015a). Because of the lifestyle of the host for water filtration, sponge symbionts are also exposed to the microenvironment with high-flux viruses (Pascelli et al., 2020). In 1978, transmission electron micrographs revealed the presence of viral-like particles (VLPs) in sponges (Vacelet and Gallissian, 1978). Viral ecology analysis in nine sponge species from the Great Barrier Reef and seven from the Red Sea sponges has provided a comprehensive insight into sponge-associated phage communities (Pascelli et al., 2020). Imaging and bioinformatics analyses indicated the importance of animal–phage–bacterium tripartite interplay in a sponge holobiont (Jahn et al., 2021). Briefly, sponge-associated viromes are becoming new research hotspots, particularly for deep-sea sponge-associated viromes.

In this study, we report an undescribed deep-sea glass sponge species from the South China Sea and provide a genome-centric view of its microbiome. We successfully retrieved 14 microbial metagenome-assembled genomes (MAGs). Among them, a novel Nitrososphaerota AOA species was found to dominate the prokaryotic consortium and exhibit typical symbiotic characteristics. We also identified two *Bdellovibrio* species that have probably undergone symbiotic evolution for adaptation to a sponge-associated lifestyle. Finally, we propose a new conceptual framework based on all possible interactions in the sponge holobiont, especially with the potential involvement of phages as we identified many phage-like contigs in the sponge metagenome.

## Materials and methods

### Sample collection and DNA extraction

The *R/V Tansuoyihao* TS-7 cruise was carried out in April 2018 in the South China Sea (SCS). A single sponge specimen (hereafter referred to as SQW35) was collected by dive No. 35 of the manned submersible *Deep-Sea Warrior* from a north SCS site (18° 41′ N, 113° 22′ E) at ~983 m depth. Upon arrival at the main deck of the *R/V*, the specimen was rinsed several times with 0.22-μm membrane-filtered seawater to remove loosely attached microbes and debris. The cleaned samples were placed into separate sterile plastic bags, transported to the laboratory, and stored at −80°C. Two pieces of tissue (~0.5 cm^3^ per piece) from an inner part of the sponge body as technological replicates (SQW35-1 and SQW35-2) were separately put into 1 ml of DNA extraction buffer (50 mM Tris–HCl, 40 mM EDTA, 500 mM NaCl, 0.75 M sucrose, pH = 8) and fully cut into tiny pieces with sterile scissors. Total suspensions of two replicates were separately subjected to DNA extraction with the PowerSoil^®^ DNA Isolation Kit (MoBio Laboratories, Carlsbad, CA, USA) following the default experimental procedure. The extracted DNA was quantified by the Qubit^®^ dsDNA HS Assay Kit with Qubit 2.0 fluorometer (Invitrogen, Carlsbad, CA, USA) and stored at −80°C for further processing.

### Metagenome DNA sequencing and prokaryotic community analyses

One hundred nanograms of genomic DNA for each replicate were randomly fragmented to ~350 bp by Covaris M220 Focused-ultrasonicator (Covaris, Massachusetts, USA). Two metagenomic libraries were constructed using the TruSeq^®^ Nano DNA LT Kit (Illumina, San Diego, CA, USA) and sequenced on a HiSeq2500 platform (Illumina, San Diego, CA, USA) to produce 2 × 150 bp paired-end reads. Raw reads were trimmed and filtered by Fastp v0.20.0 (Chen et al., 2018) with default settings. The data were further processed with FastUniq v1.1 (Xu et al., 2012) to remove duplicated reads. 16S rRNA gene sequences were predicted from clean reads using the rRNA_HMM program for analysis (subsequently referred to as 16S miTags) (Huang et al., 2009) and subjected to QIIME1 pipelines for microbial community analyses (Caporaso et al., 2010). Briefly, the 16S rRNA reads with a shared similarity of 97% were clustered to operational taxonomic units (OTUs) using UCLUST. The longest read of each OTU was selected as the representative for further taxonomic classification with the SILVA 138 database as a reference (Quast et al., 2013). OTUs that were annotated as chloroplasts, mitochondria, and eukaryotes were excluded. False positive OTUs that were not assigned to any taxa were also removed. Finally, the taxonomic relative abundance of the microbial communities in two replicates was calculated at the phylum level.

### Metagenome assembly and genome binning

Clean data of two technical replicates, SQW35-1 and SQW35-2, were separately assembled using both SPAdes v3.1.1 with the recommended settings for metagenomes (Bankevich et al., 2012) and MEGAHIT v1.2.6 with default settings (Li et al., 2015). Four assembled datasets (SQW35-1-spades, SQW35-2-spades, SQW35-1-megahit, and SQW35-2-megahit) were subjected to MetaWRAP v1.2.1 (Uritskiy et al., 2018) for genome binning with default settings, which generated 38 MAGs. These retrieved MAGs were then de-replicated together using dRep v.2.2.3 (Olm et al., 2017), which resulted in 14 high-quality MAGs. CheckM v1.0.11 was used to evaluate the completeness and contamination of the MAGs (Parks et al., 2015). Those with completeness > 50% and contamination < 10% were retained. Taxonomic annotation and relative evolutionary distance (RED) calculation of the MAGs were carried out by GTDB-tk v0.2.2 (Chaumeil et al., 2019). The nomenclature of the MAG was based on the List of Prokaryotic names with Standing in Nomenclature (LPSN) (https://lpsn.dsmz.de/).

### Relative abundance and global distribution of the dominant sponge-associated microbes

The relative abundance of a MAG was estimated as the percentage of mapped metagenomic reads with the removal of eukaryotic reads. First, metagenomic contigs with a length of >1,000 bp were subjected to EukRep v.0.6.7 to identify eukaryotic contigs (West et al., 2018). Then, the metagenomic reads were mapped to the eukaryotic contigs by Bowtie2 (Langdon, 2015), and the mapped reads were removed from the metagenomes. Finally, the remaining reads were subjected to coverM v0.2.0 (https://github.com/wwood/CoverM) to calculate the relative abundance of sponge-associated MAGs. To investigate the global distribution of a sponge symbiont, the V4 region of the 16S rRNA gene extracted from the MAG was searched by BLASTN (e-value cutoff: 1e-05) against the datasets of 16S rRNA gene sequences in the Sponge Microbiome Project (SMP) (Moitinho-Silva et al., 2017b) and the Deep-sea Sponge Microbiome Project database (D-SMP) (Busch et al., 2022). Target sequences with >96% identity were retained to calculate their relative abundance in respective sponges.

### Genome annotation

Close relatives of symbiotic MAGs were obtained by the phylogenomic analysis of the GTDB-tk program, and their genome sequences were downloaded from the NCBI and Integrated Microbial Genomes (IMG) databases. Open reading frames (ORFs) of our MAGs and reference genomes were predicted by Prodigal v2.6.2 (Hyatt et al., 2012). The predicted genes were annotated by KofamScan v1.1.0 (Aramaki et al., 2020) against KEGG databases. Eukaryotic-like protein (ELP) domains were annotated by PfamScan script against the PFAM database (El-Gebali et al., 2019). ORFs were searched by BLASTP v2.5.0 against the COG_2019_v11.0 database to find genes encoding a transposase. Clustered regularly interspaced short palindromic repeats (CRISPRs) and Cas proteins were predicted using the online CRISPRminer2, a toolkit comprising CRISPRCasFinder, CRT, and PILER-CR programs (Zhang et al., 2018). Pairwise ANI values of MAGs were calculated by PyAni v0.2.10 (Pritchard et al., 2016) with the ANIb model. AAI values of MAGs were calculated by CompareM v0.0.23 (https://github.com/dparks1134/CompareM).

### Phylogenetic analysis

The *coxI* (cytochrome c oxidase subunit I) gene of the deep-sea sponge sample was annotated from metagenome contigs by Prokka v1.13.7 (Seemann, 2014). 16S rRNA genes of symbiotic MAGs were predicted using the RNA_HMM program (Huang et al., 2009). For phylogenetic analysis of both *coxI* and 16S RNA genes, the targeted gene sequences were searched by the BLASTN program against the NCBI GenBank database to identify close relatives. The collected gene sequences were aligned using MAFFT v7.427 (Katoh and Toh, 2010) and trimmed using trimAl v1.4 (Capella-Gutiérrez et al., 2009). Phylogenetic trees were built using IQ-TREE v1.6.10 (Nguyen et al., 2015) with the “TIM2+F+I+G4” model. For phylogenomic analysis, the alignment of 43 concatenated conserved proteins deduced from our MAGs and reference genomes was produced using the CheckM program with default settings and further treated with trimAl v1.4 (Capella-Gutiérrez et al., 2009) to remove poorly aligned regions. The maximum likelihood (ML) tree was built using IQ-TREE v1.6.10 (Nguyen et al., 2015) with the “LG+F+R7” models. Bootstrap values of the trees were calculated based on 1,000 replicates.

### Identification of phage-like assembled contigs

Sponge metagenomic contigs in size of >5,000 bp were imported into DeepVirFinder (Ren et al., 2020), VIBRANT (Kieft et al., 2020), VirFinder (Ren et al., 2017), VirSorter (Roux et al., 2015b), and VirSorter2 (Guo et al., 2021) to identify viral genomes. CheckV v0.8.1 (Nayfach et al., 2021) was used to assess the viral genome quality, to identify and remove potential host contamination in integrated proviruses, and to match closely relative viral genomes from public databases. Viral genome completeness was estimated by searching against a database that comprises 76,262 complete viral genomes from publicly available metagenomes, metatranscriptomes, and metaviromes by IMG/M (Chen et al., 2019), MGnify (Mitchell et al., 2020), and the study of the human microbiome (Nayfach et al., 2019) and ocean virome (Gregory et al., 2019). Taxonomic assignment of positive viral contigs was performed using vConTACT2 (Bin Jang et al., 2019), which was designed to cluster the protein sequences with a RefSeq database based upon shared protein clusters. The relative abundance of each viral contig in a deep-sea sponge sample was calculated by mapping clean reads to assembled contigs using BWA-MEN with default settings (Li and Durbin, 2010).

## Results

### Sponge morphology and taxonomic identification

**Figure 1:**
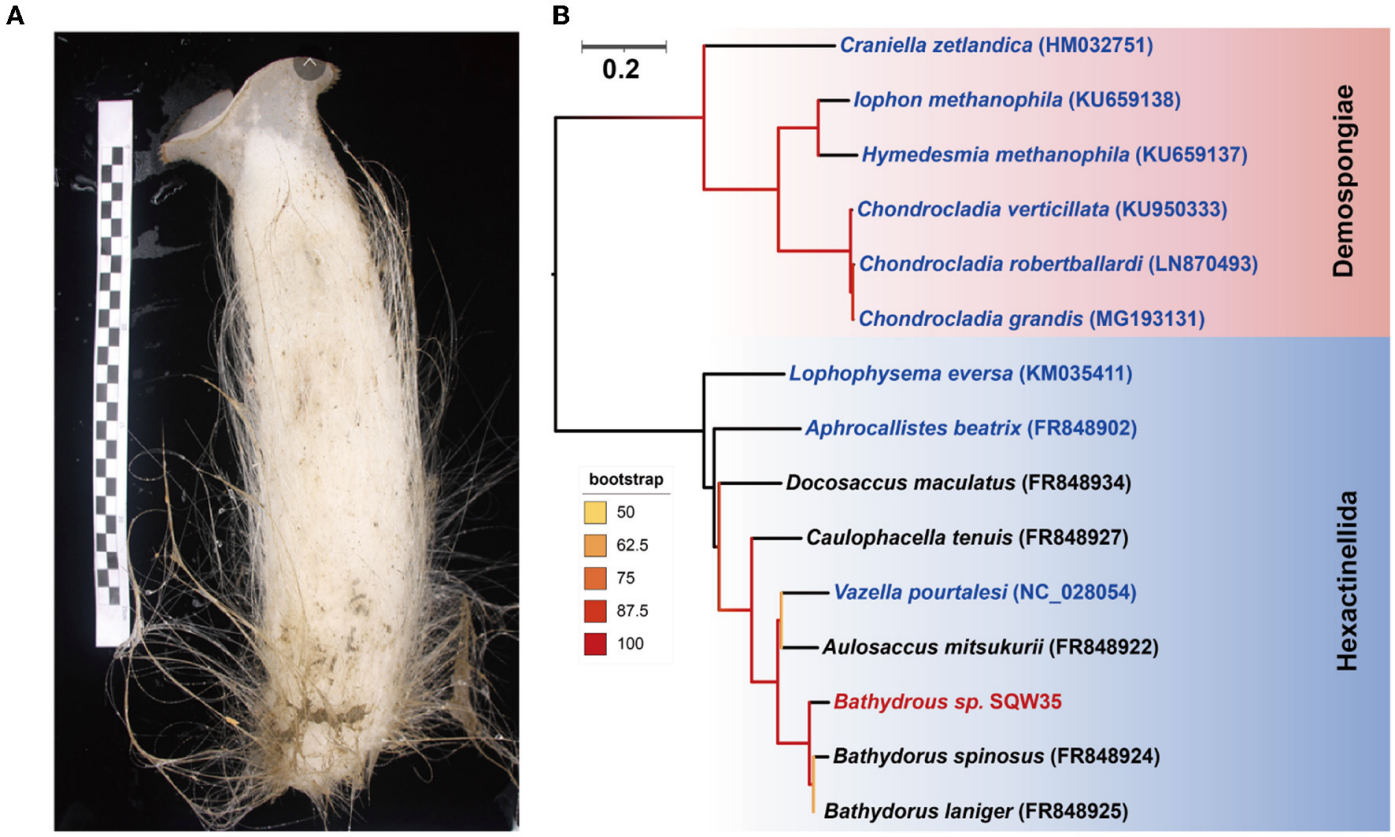
New glass sponge from the South China Sea. **(A)** A photograph of the sponge *Bathydorus* sp. SQW35. The left scale bar unit is 10 mm, and the body length is about 350 mm. **(B)** The *coxI*-based maximum-likelihood phylogenetic tree was constructed using the “TIM2+F+I+G4” model. The scale bar represents 0.2 substitutions per nucleotide position.

A thick-walled tubular deep-sea sponge was collected from the South China Sea at a depth of 983 m. The top of the sponge body is a moderately sized, trumpet-shaped osculum. The body color is pale beige. Prostalia protrudes over the body by several centimeters (Figure 1A). Phylogenetic inference based on the *coxI* gene (1,575 bp in size) suggested that the sponge is closely related to *Bathydorus laniger* from the coast of California (Kahn et al., 2013) and *Bathydorus spinosus* in the Weddell Sea (Dohrmann et al., 2011), two known species of the genus *Bathydorus* in the glass sponge class Hexactinellida (Figure 1B). The *coxI* gene of our sponge sample shared an identity of 94.83 and 93.27% with its two *Bathydorus* relatives. The *coxI* genes had respective matching thresholds at order, family, and genus levels (45, 73, and 91%, respectively) based on a sponge identification protocol proposed by a previous study based on 37 sponge species belonging to 10 orders from South Australia (Yang et al., 2017). Thus, our sample should represent a novel sponge species in the genus *Bathydorus* and is preliminarily named *Bathydorus* sp. SQW35.

### Prokaryotic composition in the sponge *Bathydorus* sp. SQW35

Taxonomic classification of 16S miTags demonstrated highly consistent prokaryotic communities in two technical replicates of the sponge *Bathydorus* sp. SQW35. The bacterial symbionts were mainly affiliated with the phyla Pseudomonadota (synonyms Proteobacteria) (Oren and Garrity, 2021), Planctomycetota, Nitrospirota, and Bacteroidota, and archaeal symbionts were dominated by the phylum Nitrososphaerota (synonyms Thermoproteota) (Supplementary Figure 1; Supplementary Table 1). Among them, the highest proportion belonged to Nitrososphaerota, which accounted for 81.81 ± 2.09% of the prokaryotic communities. Further taxonomic analysis revealed the dominance of the Nitrosopumilaceae family in Nitrososphaerota, known as AOA, which can obtain energy from ammonia oxidation and use CO_2_ as a carbon source for chemoautotrophy (Wang et al., 2019). Gammaproteobacteria that mainly belonged to SOBs were the second dominant group in the sponge *Bathydorus* sp. SQW35 and accounted for 10.60 ± 0.85% of the prokaryotic communities. Alternatively, Nitrospirota that is involved in nitrite oxidation carbon fixation (Lücker et al., 2013) occupied 0.85 ± 0.11% of the prokaryotic communities.

### Recovery of novel prokaryotic genomes

In total, 14 metagenome-assembled genomes (MAGs) with >50% estimated completeness and <10% contamination were obtained from the sponge metagenomes of the same sponge individual, and five of them were more than 90% complete (Table 1). The MAGs have a genome size ranging from 0.54 to 3.63 Mbp and GC content of 31.93–52.87%. Classification by the GTDB-tk program revealed that these MAGs were affiliated with the phyla Nitrososphaerota, Pseudomonadota, Nitrospirota, Bdellovibrionota, SAR324, Bacteroidota, and Patescibacteria (Supplementary Table 2). Relative evolutionary divergence (RED) that infers a phylogenetic distance between the last common ancestor (set to RED = 0) and all extant taxa (RED = 1) can be used to establish taxonomic ranks (Parks et al., 2018). RED values indicated that only MAG B05 fell into known species and the others likely represent novel species. Among these novel species, five MAGs have RED values of 0.71–0.89, indicative of novel genera. Three MAGs with RED values of 0.64–0.69 are suggested to present novel families (Parks et al., 2018).

**Table 1:** General genomic features of the 14 sponge-associated prokaryotic MAGs.

**MAG**	**Phylum^a^**	**Genome size (Mbp)**	***N_50_*** **(kb)**	**No. of contigs**	**No. of CDSs**	**% GC**	**Compl. (%)^b^**	**Contam. (%)^b^**	**RED^c^**
B01	Nitrososphaerota	1.45	0.23	100	1,692	33.92%	98.54	0	0.87
B02	Nitrososphaerota	1.61	0.21	124	2,027	33.00%	99.03	0.97	0.98
B03	Nitrososphaerota	1.00	0.47	37	1,206	31.93%	75.73	0.07	0.99
B04	Nitrososphaerota	1.65	0.28	97	2,119	32.16%	95.63	2.91	0.99
B05	Nitrososphaerota	0.54	0.04	145	758	35.34%	56.15	0.97	N/A
B06	SAR324	1.49	2.84	10	1,321	40.31%	87.96	0	0.51
B07	Pseudomonadota	1.09	1.27	16	1,054	44.39%	84.62	0	0.64
B08	Nitrospirota	3.63	0.14	358	4,177	49.76%	97.67	1.82	0.96
B09	Bacteroidota	3.61	0.04	1,017	3,515	35.26%	62.46	1.76	0.69
B10	Patescibacteria	0.44	0.09	61	478	32.72%	69.49	0	0.74
B11	Bdellovibrionota	1.24	0.96	17	1,144	32.44%	90.12	0	0.80
B12	Bdellovibrionota	1.67	0.05	343	1,741	35.41%	86.14	2.68	0.67
B13	Pseudomonadota	1.88	0.13	165	1,908	52.87%	79.49	4.69	0.71
B14	Pseudomonadota	1.62	0.38	73	1,576	35.34%	89.94	0.61	0.89

a The nomenclature of the MAG was based on the List of Prokaryotic names with Standing in Nomenclature (LPSN) (https://lpsn.dsmz.de/).

b Genome completeness (Compl.) and contamination (Contam.) were estimated by CheckM (Parks et al., 2015).

c RED, relative evolutionary divergence indicates taxonomic novelty (Chaumeil et al., 2019).

**Figure 2:**
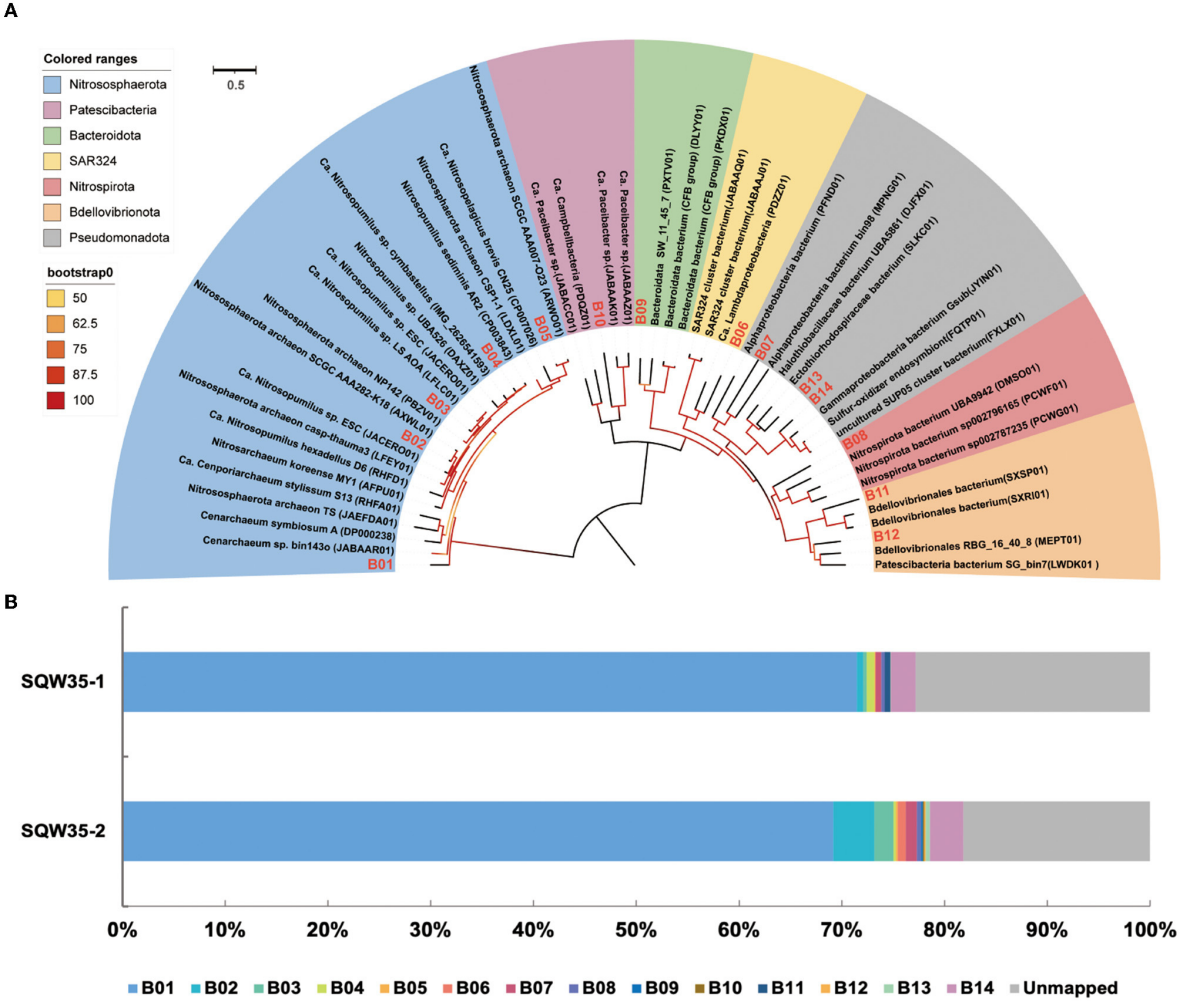
Phylogenomic tree of sponge-associated MAGs and their relative abundance. **(A)** Phylogenomic maximum-likelihood tree of the 14 sponge-associated MAGs was constructed using the “LG+F+R7” model. Sponge-associated MAGs of *Bathydorus* sp. SQW35 are marked with red in the tree. All the reference genomes are listed in Supplementary Table 12. **(B)** Relative abundance of sponge-associated MAGs in the metagenomes of *Bathydorus* sp. SQW35. The relative abundance of MAGs was calculated with coverM v0.2.0 using metagenome reads after eliminating reads assigned to eukaryotic contigs.

Phylogenomic inference also revealed that the 14 symbiotic MAGs fell into seven phyla, and the far distance of these MAGs with known species further indicated their taxonomic novelty (Figure 2A). Five MAGs (B01–B05) fell into the clade composed of AOAs in the phylum Nitrososphaerota, whereas only B03 was closely related to sponge-associated AOAs. ANIs between B03 and its close relatives *Ca*. Nitrosopumilus sp. ESC from the sponge *Hymedesmia (Stylopus) methanophila* (Haber et al., 2020) and *Ca. Nitrosopumilus* sp. LS from the glass sponge *Lophophysema eversa* (Tian et al., 2016) were 90.40 and 90.86%, respectively (Supplementary Table 3). The affiliation of MAG B14 with sulfur-oxidizing Gammaproteobacteria Gsub from the cold seep sponge *Suberites* sp. (Tian et al., 2017) in the phylogenomic tree was in line with their ANI of 70.80%. Nitrite-oxidizing bacterium (NOB) MAG B08 in the phylum Nitrospirota was affiliated with the genus SPGG5 and was placed together with Nitrospinae bacterium UBA9942 (Parks et al., 2018), which shared an ANI of 67.37%. MAGs B12 and B11 were classified as the phylum Bdellovibrionota and shared the ANIs of 68.07 and 67.91%, respectively, with its relatives Bdellovibrionales RBG_16_40_8 (Kauffman et al., 2018) and Bdellovibrionales bacterium SXSP01 (Xing et al., 2020).

The relative abundances of the sponge symbionts in metagenomes of the sponge *Bathydorus* sp. SQW35 are summarized in Figure 2B. The AOA MAG B01 recruited 70.31 ± 1.15% of the prokaryotic metagenome reads and represented the sole dominant symbiont (Figure 2B; Supplementary Table 4). The SOB MAG 14 as the second most abundant symbiont was mapped by 2.83 ± 0.40% of the prokaryotic metagenomic reads. The NOB MAG B08 could also be mapped with less abundant metagenomic reads. Relative abundances of the representative MAGs including AOA B01, SOB B14, and NOB B08 were consistent with the result of the 16S miTags analysis. In contrast, the Bdellovibrionota MAGs B11 and B12 that accounted for 0.29 ± 0.29% and 0.07 ± 0.07% metagenomic reads were not detected in 16S miTags analysis.

### A specialist sponge AOA symbiont lineage

**Figure 3:**
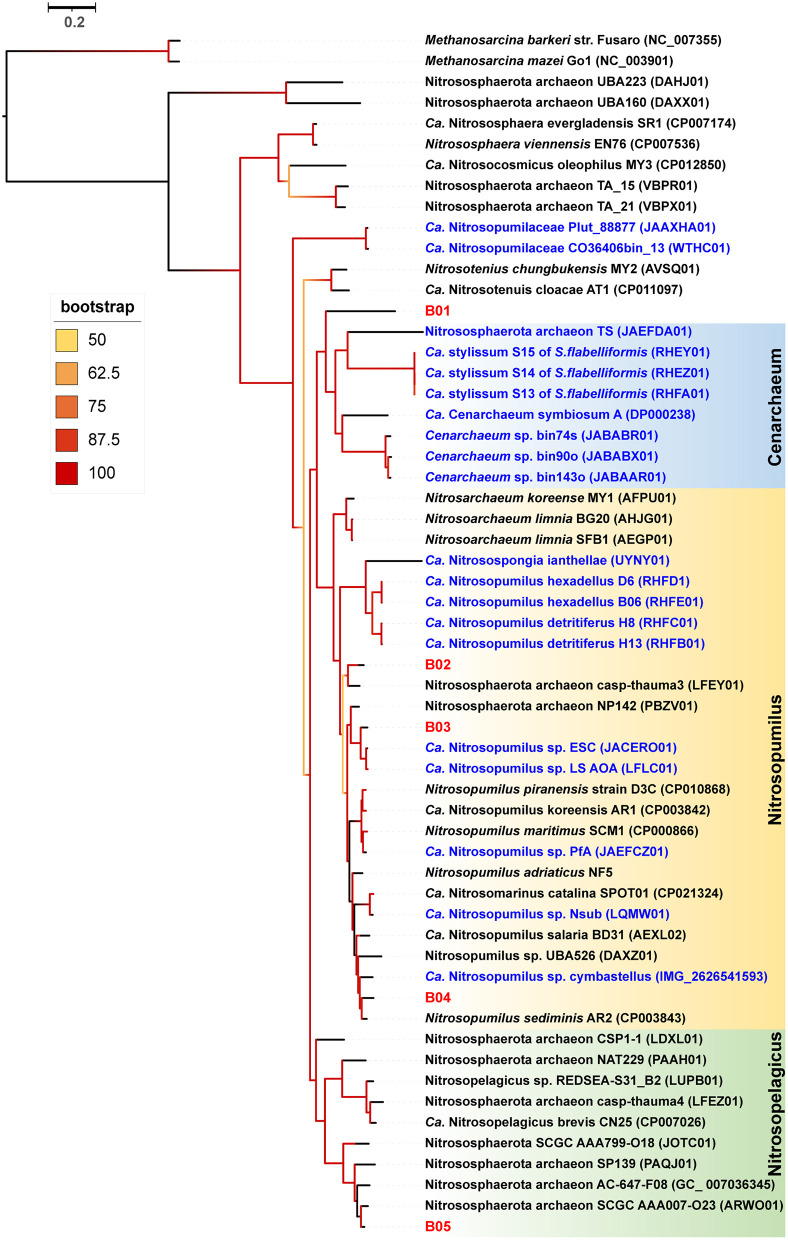
Phylogenomic tree of the sponge-associated AOA MAGs. The phylogenomic maximum-likelihood tree of sponge-associated AOA MAGs was constructed using the “LG+F+R7” model. *Bathydorus* sp. SQW35 sponge-associated MAGs are marked with red. Sponge-associated reference MAGs from other environments are marked with blue. *Methanosarcina barkeri* and *Methanosarcina mazei* Go1 (p_Euryarchaeota) are used as outgroups. All the reference genomes are listed in Supplementary Table 12.

**Figure 4:**
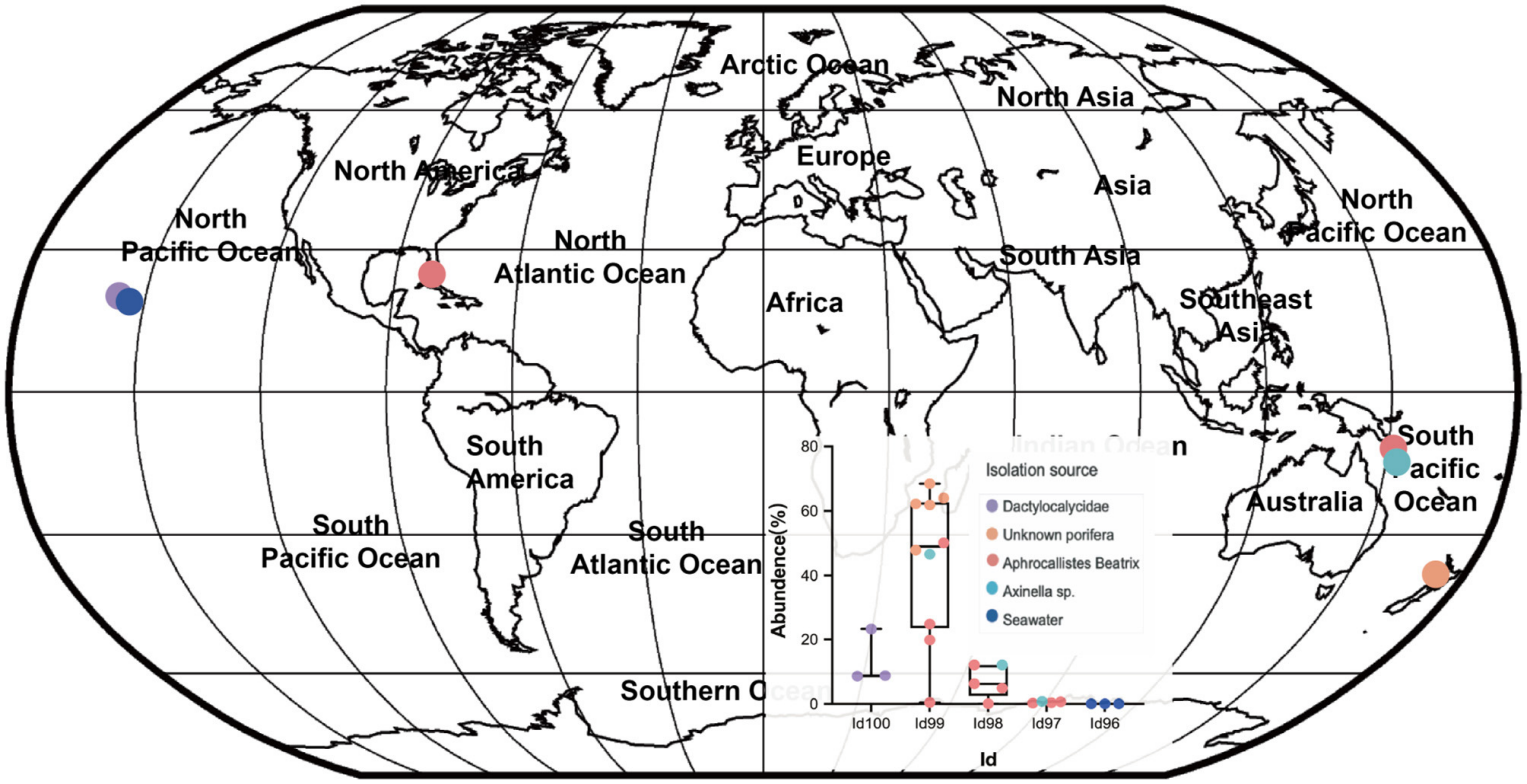
Global distribution of the sponge symbiont Nitrososphaerota B01. To investigate the distribution of B01 in sponges and surrounding environmental samples, its 16S rRNA gene sequence as a query was searched against the Sponge Microbiome Project (SMP) using BLASTN, and the target sequences with identity values of >96% were selected and assigned to Id100 (identity = 100%), Id99 (identity ≥ 99% but < 100%), Id98, Id97, and Id96 groups. The relative abundance of the B01 relatives in the SMP samples was calculated. Target sponge species and environmental samples were labeled on the world map.

Our detailed phylogenomic inference revealed that the dominant symbiont AOA B01 was closely related to the *Cenarchaeum* clade that is now composed of four lineages including *Cenarchaeum symbiosum* A from the demosponge *Axinella mexicana, Cenarchaeum* sp. bin74s, *Cenarchaeum* sp. bin90o, and *Cenarchaeum* sp. bin143o (phylum: Nitrososphaerota) from *Vazella pourtalesii*, Nitrososphaerota archaeon TS (phylum: Nitrososphaerota) from *Theonella swinhoei*, and *Candidatus* Cenporiarchaeum stylissum S13–S15 (phylum: Nitrososphaerota) from *Stylissa flabelliformis* (Figure 3). ANIs between B01 and these reference genomes range from 68.96 to 69.97% (Supplementary Table 3). With its RED value of 0.87 (Table 1), MAG B01 should represent a new genus. Global distribution analysis against the SMP database (Moitinho-Silva et al., 2017b) revealed that the V4 region of the 16S rRNA gene in MAG B01 showed 100% identity with amplicons from three glass sponge individuals in the family Dactylocalycidae (Figure 4), with relative abundances of 23.25, 8.78, and 8.60% in the respective communities (Supplementary Table 5). B01 also shared 97–99% identities with sponge symbionts in three other sponge species, *Aphrocallistes beatrix, Axinella* sp., and an unnamed species, but was only 96% similar to its closest relatives from seawater samples (Supplementary Table 5). By a query against the D-SMP database (Busch et al., 2022), B01 shared more than 99% identity with symbionts in *Pheronema carpenteri, Bathydorus* sp., *Aphrocallistes beatrix, Saccocalyx tetractinus, Rossellinae indet*, and *Lophocaly*x sp. The relative abundances of B01 in these sponges span between 0.007 and 0.657% (Supplementary Table 5). Because the D-SMP database employed bacterial primers for amplification, the relative abundance of archaeal relatives of MAG B01 in this database is likely underestimated. Sponge symbionts can be classified into generalists (found in a wide range of sponge species) and specialists (living in a small number of sponge species) according to their distribution patterns (Haber et al., 2020). In brief, the distribution patterns against both the SMP and D-SMP databases indicate that AOA B01 is a specialist.

### Bdellovibrionota predators as sponge symbionts

**Figure 5:**
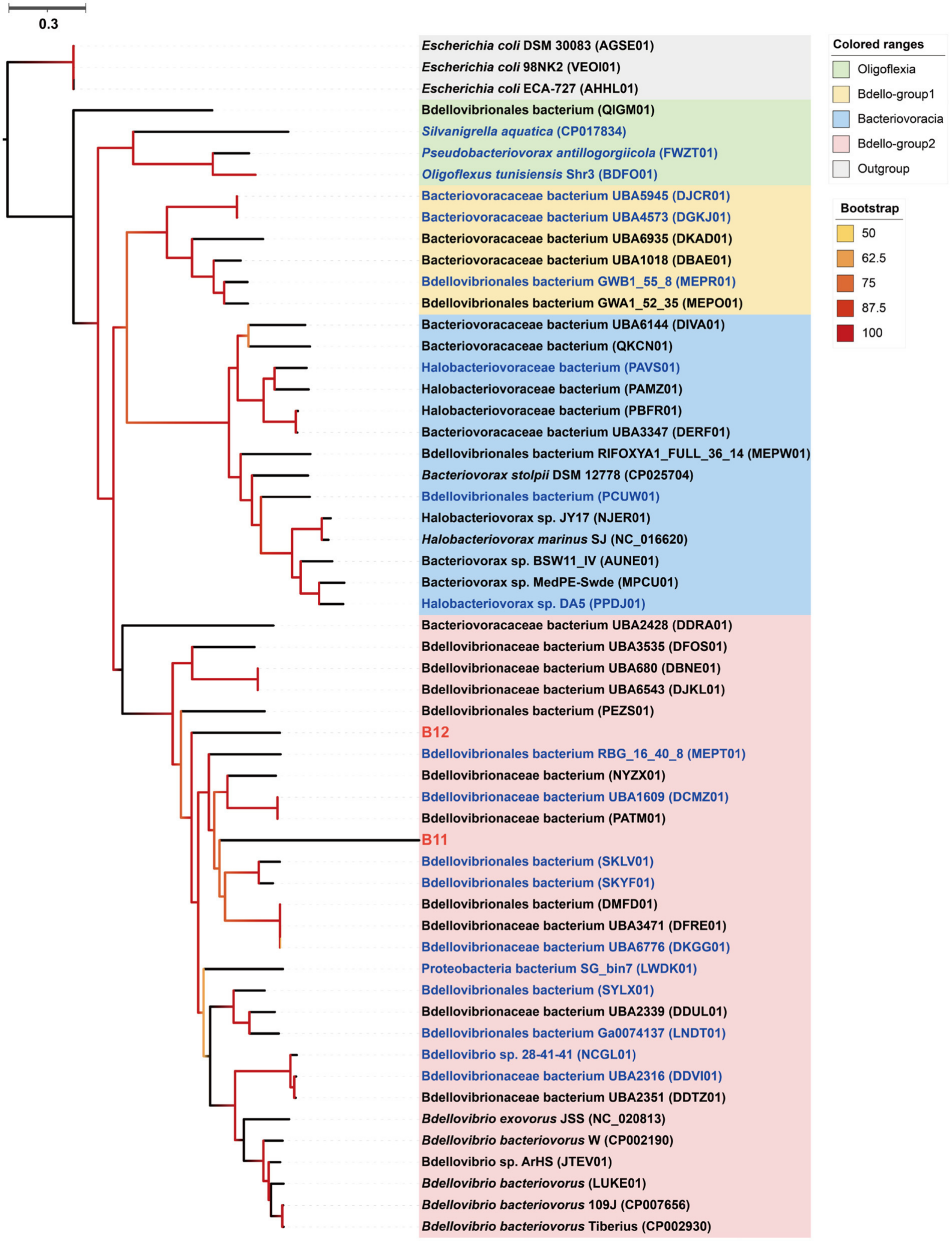
Phylogenomic tree of the sponge-associated Bdellovibrionota MAGs. The maximum-likelihood tree was constructed using the “LG+F+R7” model. *Escherichia coli* DSM 30083, *Escherichia coli* 98NK2, and *Escherichia coli* ECA-727 were used as outgroups. Sponge-associated Bdellovibrionota MAGs B11 and B12 were colored in red. Free-living Bdellovibrionota MAGs with completeness > 90% were colored in blue.

Phylogenomic inference showed that both MAGs B11 and B12 fell in the Bdello-group2 in the phylum Bdellovibrionota but form two separate deep branches (Figure 5). Bdello-group2 comprised of the representative *Bdellovibrio* predators including *Bdellovibrio exovorus* JSS, *Bdellovibrio bacteriovorus* HD100, *Bdellovibrio bacteriovorus* Tiberius, and *Bdellovibrio bacteriovorus* 109J (Li et al., 2021), suggesting that B11 and B12 may also represent bacterial predators. The Bdellovibrionota MAGs B11 and B12 have remarkably smaller genome sizes and lower GC contents than those free-living Bdellovibrionota species (Supplementary Figure 2). The comparative genomic analysis further revealed that the free-living Bdellovibrionota bacteria harbored genes encoding chemotaxis proteins and mobility systems (flagellum and type IV pili), yet MAGs B11 and B12 lacked these genes (Supplementary Table 6). Predatory *Bdellovibrio* bacteria invade the periplasm of bacterial prey cells by penetrating the peptidoglycan layer to form transient structures there. Penicillin-binding proteins (PBPs) are important for Bdellovibrionota to lyze the cell wall of a prey bacterium (Lerner et al., 2012). Annotation analysis showed that symbiotic Bdellovibrionota MAGs lack the PBP1C coding gene compared to free-living relatives but retain PBP1A, PBP1B, PBP2, PBP3, and PBP4 coding genes (Supplementary Table 6), which suggests that MAGs B11 and B12 may still retain the predatory potential.

### Carbon, nitrogen, and sulfur metabolisms of the sponge symbionts

We searched the three main prokaryotic autotrophic carbon fixation pathways: 3-hydroxypropionate/4-hydroxybutyrate (3HP/4HB) cycle, reductive citric acid (rTCA) cycle, and the Calvin–Benson–Bassham (CBB) cycle. The 3HP/4HB cycle was identified in sponge-associated AOA (family *Nitrosopumilaceae*) with the presence of key genes encoding 3-hydroxypropionyl-CoA synthetase (K18594) and 4-hydroxybutyrate-CoA ligase (K18593) (Supplementary Table 7). Almost all genes of the rTCA cycle were identified in Nitrospirota B08 (family UBA8639, genus SPGG5), including those coding for citrate synthase (K01647), fumarate hydratase (K01676), and succinate dehydrogenase (K00240), which were considered as evidence for the rTCA cycle. Furthermore, more than 90% of the genes for carrying out the CBB cycle including those encoding subunits of ribulose-bisphosphate carboxylase (K01602 and K01601) were found in the SOB MAGs (class Gammaproteobacteria). These findings indicate the existence of various carbon fixation pathways in the microbial consortium of the sponge *Bathydorus* sp. SQW35.

The *amoABC* genes (K10944, K10945, K10946) encoding ammonia monooxygenase subunits were identified in three Nitrososphaerota MAGs (B01, B04, and B05) but not in any bacterial MAGs (Supplementary Table 7). This suggests that AOA is the only symbiotic group in charge of ammonia oxidation in the sponge *Bathydorus* sp. SQW35. Nitrospirota B08 had the gene encoding nitrate/nitrite transporter (K02575) but lacked genes encoding nitrite oxidoreductase. In addition, the B08 has a gene set involving urea decomposition that produces ammonia for nitrification and biomass production, where urea would be imported from the extracellular milieu by the urea ABC transporter urtABCDE (K11959, K11960, K11961, K11962, and K11963) and be hydrolyzed by urease ureABC (K01428, K01429, and K01430) (Supplementary Table 7). These results further imply the potential role of NOB B08 in urea utilization.

Microbial sulfur oxidation is frequently reported in sponges to remove toxic sulfide produced by other bacterial symbionts that use sulfate as an electron acceptor (Tian et al., 2014). Gammaproteobacteria SOB B13 and B14 encode adenylyl-sulfate reductase A (K00394), adenylyl-sulfate reductase B (K00395), and sulfate adenylyltransferase (K00958) to oxidize sulfite to sulfate (Supplementary Table 7). SOB B14 also contains the *soxAXYZ* gene clusters (K17222, K17223, K17226, and K17227) that code for the SOX complex, confirming potential capacity for sulfur oxidation (Supplementary Table 7). Previous studies showed that taurine is a natural product of sponges, and sponge symbionts can import and utilize taurine as suggested by the presence of ABC transporter genes (*tauABC*) (Emura et al., 2006; Karimi et al., 2018). The SOB MAGs B13 and B14 also have genes encoding taurine ABC transporter (*tauAC*; K15551 and K15552), taurine-pyruvate aminotransferase (K03851), and sulfoacetaldehyde acetyltransferase (K03852), which can catalyze taurine to sulfite for further oxidation by the SOX complex.

### Eukaryotic-like proteins encoded by the sponge symbionts

We analyzed the distribution of ELPs including ankyrin repeats (Ank), tetratricopeptide repeats (TPRs), NCL-1, HT2A and Lin-41 repeats (NHL), fibronectin type III (Fn3) and cadherin (CAD) CUB, bacterial Ig-like domain (Big), WD40, and pyrroloquinoline quinone repeat (PQQ) in the deep-sea glass sponge microbiome (Supplementary Table 8). We found that five of the nine bacterial MAGs encoded Ank domains, including SOB B14 and NOB B08. Notably, Bdellovibrionota B11 has 12 Ank domains, which was the highest number among these bacterial MAGs. However, none of the AOA MAGs had Anks. The bacterial and archaeal MAGs, except B10, had TPR domains, especially in B08 and B09. As the most prevalent symbiont, AOA B01 encoded the maximum number of NHL domains. NOB B08 also showed the richness of NHL domains (*n* = 13).

### CRISPR–Cas systems in the sponge symbionts

One major strategy of prokaryotic defense against phages is dependence on the Clustered Regularly Interspaced Short Palindromic Repeats (CRISPR)–Cas system (Makarova et al., 2013). Here, all the MAGs were analyzed for their CRISPR–Cas systems. Almost all the symbiotic MAGs in the sponge *Bathydorus* sp. SQW35 has genes encoding Cas proteins (Supplementary Figure 3A). CRISPRminer2 analysis further indicated that CRISPR arrays could be found in six MAGs (B01, B08, B09, B11, B13, and B14), which represented almost all the dominant microbial groups (Supplementary Figure 3B; Supplementary Table 9). Interestingly, the dominant symbiont AOA B01 has a CRISPR array with the highest number of spacers (*n* = 85). The second abundant symbiont SOB B14 also has a CRISPR array with 81 spacers, followed by a CRISPR array in Bdellovibrionota B11 consisting of 51 spacers.

### Phage diversity of the sponge microbiome

To understand the high prevalence of CRISPR–Cas systems in the dominant symbiotic inhabitants, we analyzed the phages in the deep-sea glass sponge *Bathydorus* sp. SQW35. In total, 125 contigs in SQW35-1 and 148 contigs in SQW35-2 were predicted as potential phage genomes (>5,000 bp) (Supplementary Table 10). The potential viral contigs of SQW35-1 and SQW35-2 were predicted to harbor 237 and 236 viral genes by CheckV (Nayfach et al., 2021), respectively. There are 232 positive virus sequences after redundancy removal of the contigs, which indicated a large phage community in the sponge *Bathydorus* sp. SQW35. Among them, the most abundant phage fragment accounted for 1.48 and 2.19% of metagenome reads, respectively, in the two replicate samples (Supplementary Table 11).

## Discussion

While shallow-water sponge holobionts have been extensively studied in the last two decades, our knowledge about deep-sea sponges and their associated microbes remains rare. The present study collected a new deep-sea sponge species affiliated with the genus *Bathydorus* (*Bathydorus* sp. SQW35) from the South China Sea. Both 16S miTags-based prokaryotic community analysis and relative abundance analysis of MAGs demonstrated an AOA-dominant microbiome. AOAs together with SOBs and NOBs comprise the key prokaryotic players involved in carbon, sulfur, and nitrogen metabolism in the sponge holobiont, which is consistent with previous findings of prokaryotic communities in a large number of deep-sea sponge species (Busch et al., 2022). In total, 14 prokaryotic MAGs were successfully retrieved from the sponge microbiome, and 13 MAGs belong to potential new species, indicating the novelty of prokaryotic genome resources in deep-sea sponges (Wang et al., 2022).

Ammonia oxidation plays a significant role in the nitrogen cycle in marine. AOAs in the phylum Nitrososphaerota have key roles in the global nitrogen cycle and are also widely found in sponge holobionts (Moitinho-Silva et al., 2017a; Zhang et al., 2019b; Steinert et al., 2020). *Cenarchaeum symbiosum* A is the first reported sponge AOA symbiont in the family *Nitrosopumilaceae* (Hallam et al., 2006). Recent genomic work has expanded the *Cenarchaeum* clade to four lineages (Zhang et al., 2019b; Bayer et al., 2020; Haber et al., 2020). Till now, this clade has only been reported in sponge holobionts and likely represents a sponge-specific cluster (Preston et al., 1996; Schleper et al., 1998; Hallam et al., 2006; Bayer et al., 2020). The dominant AOA MAG B01 of *Bathydorus* sp. SQW35 was closely related to the *Cenarchaeum* clade but probably represents a novel species in a new genus, which thus discloses the 50 lineages of the *Cenarchaeum* clade and expands the diversity of sponge AOA symbionts. This finding of novel Nitrososphaerota symbionts helps to uncover unique adaptation mechanisms of AOAs to sponge hosts. Sponges can excrete ammonia as a metabolic waste, which produces an ammonia-rich microenvironment in their bodies, and thus benefits the nitrification process (Moitinho-Silva et al., 2017a). AOA B01 is the sole dominant symbiont of *Bathydorus* sp. SQW35 and its relative abundance are much higher than those AOAs associated with shallow-water and even deep-sea sponges. Thus, AOA B01 likely plays a much more important role in ammonia oxidization in this deep-sea sponge species. Meanwhile, the dependency of this deep-sea sponge on ammonia oxidization for ammonia elimination and nutrition supply is likely much higher than shallow-water sponges.

*Bdellovibrio* (in the phylum Bdellovibrionota) and *Bdellovibrio*-like bacteria (BALOs) are gram-negative bacterial predators living in various environments (Jurkevitch et al., 2000; Sockett, 2009) and mainly consists of four groups: Bacteriovoracia, Oligoflexia, Bdello-group1, and Bdello-group2 (Li et al., 2021). Till now, sponge-associated BALO had only been reported in the shallow-water sponge *Cymbastela concentrica*, and this predator was proposed to live with cyanobacteria (Tian et al., 2017). The MAGs B11 and B12 of the sponge *Bathydorus* sp. SQW35 are affiliated with the Bdello-group2 in the Bdellovibrionota, providing additional examples of BALOs with a possible role of sponge symbionts. Genomic reduction is common for microbial symbionts when some genes are no longer required during adaptive evolution (McCutcheon and Moran, 2011). Low GC content correlates with symbiotic evolution because of mutational bias in symbionts (Wernegreen and Funk, 2004; Bohlin et al., 2020). The smaller genome size and lower GC content of MAGs B11 and B12 compared to their free-living relatives also support their symbiotic lifestyle indicative of reductive genome evolution. Motility machines are needed for the free-living predatory Bdellovibrionota to capture the bacterial prey (Lambert et al., 2009). The loss of genes encoding motility machines in MAGs B11 and B12 indicates that the sponge Bdellovibrionota symbionts do not need motility after their symbiotic evolution. Although the presence of PBP genes involved in predator roles suggests the predatory potential of both MAGs B11 and B12, their predator roles and prey target in the sponge *Bathydorus* sp. SQW35 remains unclear and needs further exploration.

Eukaryotic-like protein coding genes in prokaryotic genomes are markers of symbionts with a long history of coevolution with their hosts (Cazalet et al., 2004). ELPs such as Anks and TPRs are commonly enriched in sponge-associated metagenomes and are perceived to be important for the interaction between symbionts and their hosts and for helping the symbiont to avoid decomposition by the host (Díez-Vives et al., 2017). Heterologous expression of Anks from sponge symbionts has even been proven to be capable of avoiding phagocytosis of amoeba (Nguyen et al., 2014). Most of the bacterial MAGs including Bdellovibrionota B11 and SOB B14 encoded Anks, speculating their symbiotic role in *Bathydorus* sp. SQW35. Consistent with previous studies (Zhang et al., 2019b; Haber et al., 2020), none of our AOA MAGs encoded Anks; thus, their symbiotic interaction mechanism with the host would be different. TPRs mediate bacterium–eukaryote interactions and have been revealed to allow entry of *Legionella pneumophila* into epithelial cells and to regulate exopolysaccharide biosynthesis in *Pseudomonas aeruginosa* (Mittl and Schneider-Brachert, 2007). NHL was previously found to be enriched in sponge metagenomes (Fan et al., 2012), which were found in serine/threonine protein kinases and were indicated to affect phagosome processing in sponge symbionts (Reynolds and Thomas, 2016). The distribution patterns of these ELPs are discrepant among symbiotic MAGs of *Bathydorus* sp. SQW35, suggesting that symbionts employed different strategies to interact with their hosts.

Phages act as important prokaryotic killers in the oceans. They dominate seawater viral communities and lyse 20–50% of marine surface bacteria per day (Fuhrman, 1999). Sponges are likely exposed to high-concentration phages because of their filtration activity (Pascelli et al., 2020). Recent studies have illustrated diverse phage sponge-associated phage communities and tripartite sponge–phage–bacterium interplays (Pascelli et al., 2020; Jahn et al., 2021; Carrier et al., 2022). Our study also showed the involvement of diverse phages in the microbiome of the deep-sea sponge *Bathydorus* sp. SQW35. According to the virome analysis of coral and sponge-associated viruses, the virome communities were dominated by double-stranded DNA (dsDNA) bacteriophage of the order Caudovirales and a diverse community of single-stranded DNA (ssDNA) viruses of the family Microviridae (Laffy et al., 2018). However, our deep-sea sponge-associated phages could not be identified as any known viral taxa, and all predicted phage proteins were unknown in function. We suppose that deep-sea sponges contained viruses and phages of unknown taxonomy, which prompts us to investigate with further efforts. Because no reference seawater samples were analyzed in parallel, it must be declared that those phages may come from the ambient seawater.

For potential prokaryotic defense strategy against phages, genomic analyses highlight the distribution of CRISPR–Cas systems in 14 symbiotic MAGs. The dominant symbiont AOA B01 has the highest spacers (*n* = 85), and the SOB B14 comes ranks second (*n* = 81). A previous study has shown that containing more spacers in CRISPR–Cas systems could maximize the prokaryotic cell survival rate (Martynov et al., 2017). The highly complex CRISPR array might help the sponge symbionts gain a competitive advantage because of their efficient defense against phages. These findings suggest that symbiont defense mechanisms have evolved in the context of the sponge holobiont to maintain their dominant symbiont status.

**Figure 6:**
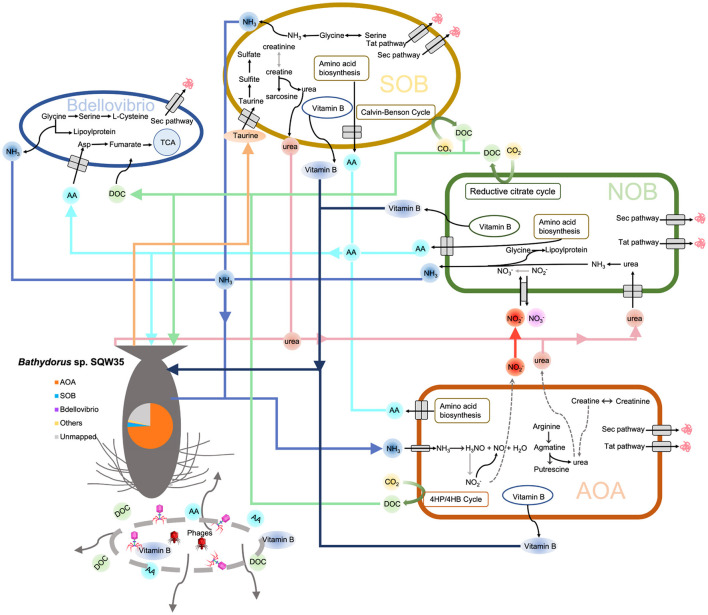
Metabolic diagram of the symbiotic consortium in *Bathydorus* sp. SQW35. Solid lines refer to the metabolic pathways in which >50% of genes/enzymes were identified, and dash lines indicate missing key genes/enzymes. The pie chart shows the relative abundance of the main symbiotic MAGs in the sponge microbiome.

Based on the earlier findings, we propose a metabolic network of the microbial consortium in the sponge *Bathydorus* sp. SQW35 (Figure 6). Sponges could obtain nutrients by feeding on both exogenetic carbon sources (Wilkinson, 1983) and autotrophically fixed carbon by their microbial symbionts such as AOAs, NOBs, and SOBs. By autotrophic CO_2_ fixation, symbionts of the deep-sea sponge provide the necessary primary metabolites and continuously stable carbon sources that enabled the sponge host to adapt to the diverse deep-sea environment. Compared with the AOAs, NOBs, and SOBs that are involved in the elimination of toxic ammonia and sulfide waste produced by the sponge, *Bdellovibrio* predators acquire organic carbons from the autotrophies and/or sponge host for a parasitic lifestyle. In addition, phages can probably infect all the sponge symbiotic inhabitants and break down the cells to release organic carbon to nourish the sponge host. This might also regulate the population size of the microbial inhabitants in sponges as suggested previously (Jahn et al., 2021).

## Conclusion

In this study, we report a novel deep-sea glass sponge species *Bathydorus* sp. SQW35 inhabiting the South China Sea and describe an AOA-dominant sponge microbiome. We uncovered a previously undescribed AOA species B01 dominating the microbiome and highlighted the Bdellovibrionota predators B11 and B12 as sponge symbionts undergoing reductive genome reduction. We further revealed the roles of CRISPR–Cas systems and ELPs in adaptive evolution to the deep-sea sponge holobiont and the involvement of phages in the symbiotic network. In summary, our results explored the symbiotic diversity, evolutionary adaptation, and symbiotic network in a deep-sea sponge holobiont. This cumulative knowledge base is a reference for subsequent research on the origin of deep-sea life. However, because of sampling limitations for deep-sea inhabitants and environment samples, we do not have multiple sponge individuals and ambient seawater and sediment samples. Whether all the analyzed microbes and phages are inherent to the sponge *Bathydorus* sp. SQW35 requires further investigations with regard to the distribution and generalization of both virome and prokaryotic microbiome.

## Data availability statement

Metagenome data and MAGs obtained for the deep-sea sponge *Bathydorus* sp. SQW35 have been submitted to the NCBI database under BioProject PRJNA871057.

## Author contributions

Z-MG and YW conceived the project. Z-MG and T-SW designed the experiments. L-SH collected the samples and took photos. LG described and identified the sponge samples. T-SW and H-GC extracted the DNA and performed the analyses. Q-ML and Y-LZ analyzed the data. T-SW drafted the manuscript. All authors edited the manuscript and approved its submission.
