# Supplementary material for: Genome-centric view of the microbiome in a new deep-sea glass sponge species Bathydorus sp
Source: Front Microbiol. 2023 Feb 8;14:1078171. doi: 10.3389/fmicb.2023.1078171 (PMC9944714; doi:10.3389/fmicb.2023.1078171)
Supplement: Supplementary file 2 [file Data_Sheet_1.docx]

**Supplementary figures:**


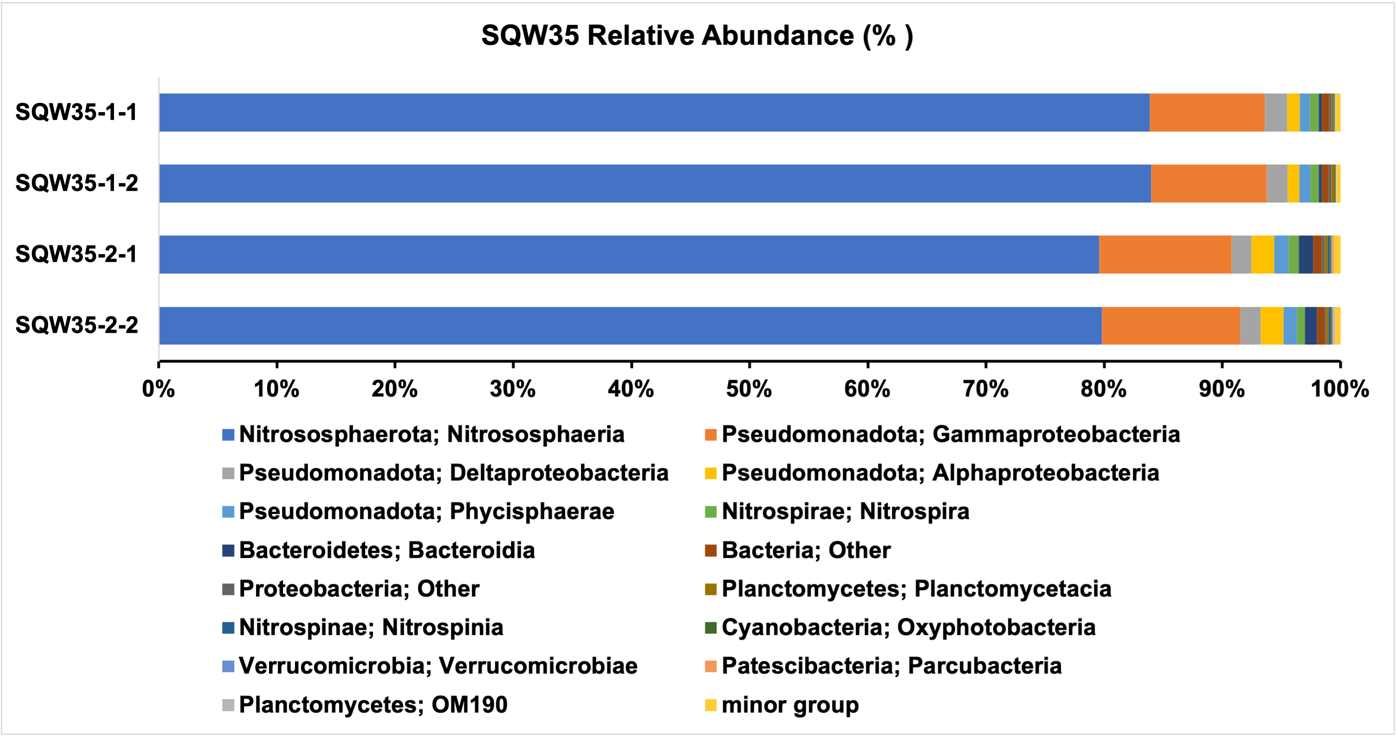


**Supplementary Figure 1.** Mitags-based relative abundance of prokaryotes in *Bathydorus* sp. SQW35. 16S rRNA MiTags were retrieved from sponge metagenomes and used for analyzing the prokaryotic composition by QIIME1.


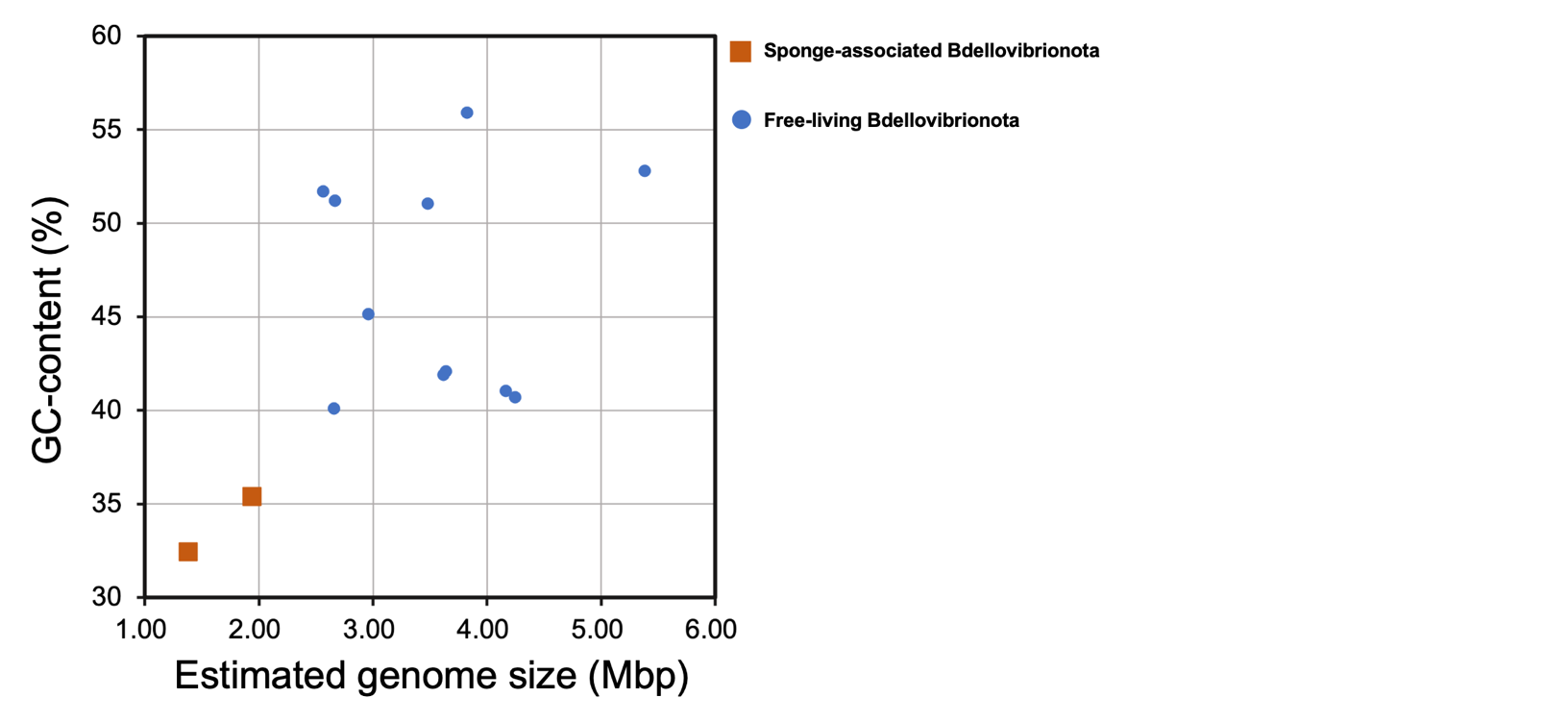


**Supplementary Figure 2.** Genome length versus GC content for sponge-associated and free-living microbes in the phylum Bdellovibrionota.


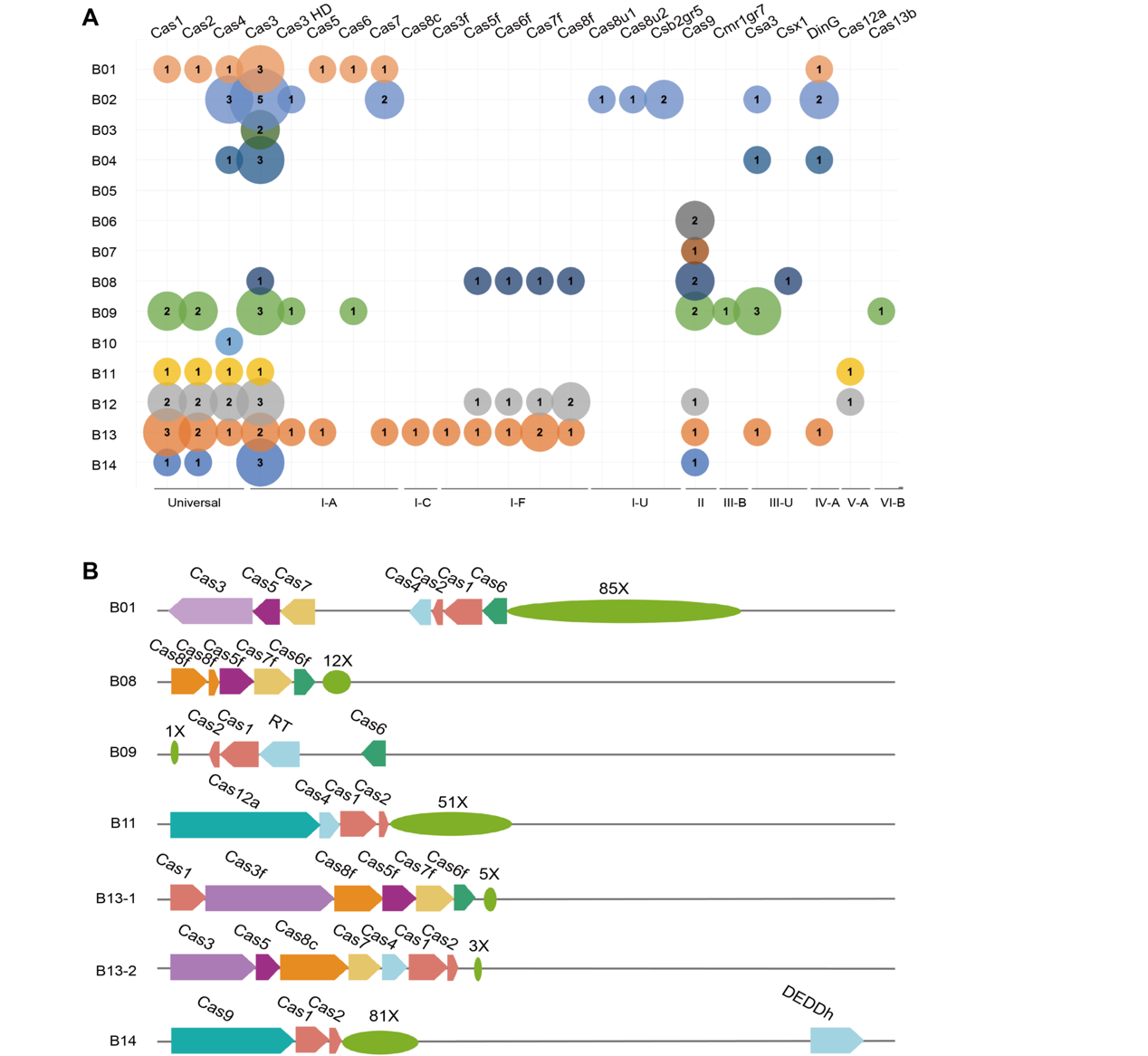


**Supplementary Figure 3.** CRISPR-Cas systems in the 14 sponge-associated prokaryotic MAGs.

(A) Number of CRISPR-associated (cas) proteins in the 14 prokaryotic MAG. (B) Schematic overview of complete CRISPR-Cas system in MAGs. The same color arrow indicates the same *cas* genes with the length of the arrow representing the length of the *cas* gens; the oval-shaped frames indicate CRISPR arrays and the upper number represents the number of repeats. CRISPR-Cas proteins and systems were annotated by CRISPRminer2 (<http://www.microbiome-bigdata.com/CRISPRminer2/index/>).
